# Microneedle-Assisted Percutaneous Delivery of a Tetramethylpyrazine-Loaded Microemulsion

**DOI:** 10.3390/molecules22112022

**Published:** 2017-11-21

**Authors:** Qiang Zu, Yanyan Yu, Xiaolin Bi, Ren Zhang, Liuqing Di

**Affiliations:** 1 School of Pharmacy, Nanjing University of Chinese Medicine, Nanjing 210023, China; zuqiang1975@163.com (Q.Z.); bxl77@126.com (X.B.); 2 Jiangsu Provincial TCM Engineering Technology Research Center for Highly Efficient Drug Delivery Systems (DDS), Nanjing 210023, China; 3 School of Chemical and Environmental Engineering, Shanghai Institute of Technology, Shanghai 201418, China; sshnhyyy@sit.edu.cn; 4 Shanghai Hutchison Pharmaceuticals Limited, Shanghai 200001, China; zhangren@shpl.com.cn

**Keywords:** microneedle, microemulsion, tetramethylpyrazine, skin permeation

## Abstract

This study examined the efficacy of the percutaneous delivery of a tetramethylpyrazine-loaded microemulsion (TMP-ME) on skin pretreated with microneedles (MN). The TMP-ME formulation was optimized in vitro with skin permeation experiments, using a uniform experimental design, guided by a pseudo-ternary phase diagram, in which the TMP skin permeation level and mean particle size were indices. The effects of MN pretreatment on skin permeation by TMP-ME were assessed using in vitro skin permeation, in vivo skin microdialysis, and pharmacokinetic studies in rats. The influence of MN pretreatment on the skin barrier function was evaluated by measuring the electrical resistance of rat skin before and after MN insertion. In the optimal formulation of TMP-ME, the weight percentages of Maisine^®^ 35-1 (oil phase), Labrasol^®^ (surfactant), and Transcutol^®^ P (co-surfactant) were 7%, 30% and 10%, respectively, with 1.5% TMP loading. In the in vitro skin permeation study, MN-assisted TMP-ME exhibited a two-fold increase in a 24-h cumulative TMP permeation compared with TMP-ME alone (*p* < 0.05). In the skin microdialysis study, TMP in MN-assisted TMP-ME exhibited a 1.25-fold increase in C_max_, a 0.93-fold decrease in T_max_, and a 0.88-fold increase in AUC_0–t_ (*p* < 0.05). Similarly, in the pharmacokinetic study, TMP in MN-assisted TMP-ME exhibited a 2.11-fold increase in C_max_, a 0.67-fold decrease in T_max_, and a 1.07-fold increase in AUC_0–t_ (*p* < 0.05). The percutaneous electrical resistance of rat skin before and after MN insertion was 850 ± 50 Ω/cm^2^ and 283 ± 104 Ω/cm^2^ respectively, indicating that MN dramatically compromises the skin barrier. These results suggest that MN assistance increases the skin permeation rate and the extent of percutaneous absorption of TMP-ME, and that the mechanism may involve the reversible barrier perturbation effect. The rate and extent of percutaneous absorption of TMP-ME can be significantly enhanced by MN assistance, possibly because MN causes a reversible barrier perturbation effect on skin.

## 1. Introduction

The percutaneous route is attractive for drug delivery because it facilitates a stable level of drug in the plasma, avoids the first pass effect, and has a high patient compliance. However, transdermal delivery is currently limited to potent compounds that have low molecular weights and favorable n-Octanol/water partition coefficients, due to the formidable barrier function of the stratum corneum (SC). To address this challenge, methods for enhancing passive and active skin penetration are being assiduously investigated [1]. Passive skin penetration enhancement protocols usually involve the application of nano-scale carriers, such as microemulsions, vesicles, and nanoparticles, to facilitate drug penetration through the skin [2,3,4]. Active skin penetration enhancement typically requires perturbing the reversible barrier function of the SC using mechanical force (microneedles), ultrasound (sonophoresis), high voltage pulses (electroporation), or the application of a small electrical potential difference (iontophoresis) to drive ionic drugs through hair follicles [5,6,7]. Efforts to improve skin permeation efficiency have led to combining passive and active enhancement techniques. For example, microneedles have been combined with liposomes, and iontophoresis has been applied with solid lipid nanoparticles [8,9,10].

A microemulsion (ME) is a dispersion consisting of oil, water, surfactant, and often a co-surfactant. The mixture behaves as a single optically-isotropic and thermodynamically-stable liquid solution with a droplet diameter, usually within the range of 10–100 nm [11]. MEs have been widely used as vehicles for percutaneous drug delivery due to their physical stability, their solubilizing potential for lipophilic and hydrophilic drugs, their protection of entrapped drugs from degradation, hydrolysis, and oxidation, and the skin penetration enhancement effects of their components [12,13,14].

Microneedles (MN) are fabricated with silicon, metals, or dissolving polymers, usually pyramidal in shape, with lengths ranging from 50 µm to 1000 µm, and are arrayed at densities of 100 to 1000 needles per cm^2^. MNs dramatically improve permeation efficiency by temporarily compromising the integrity of the SC, making it possible to efficiently deliver macromolecules into the skin without disrupting the dermis or causing pain. MNs have been used widely for intradermal and transdermal drug delivery [15,16,17]. Dissolving polymer MNs are used mainly for intradermal vaccination, due to their poor drug loading capacity [18]. Silicon or metal MNs are usually combined with other skin penetration enhancement techniques. Typically, skin is treated first with silicon or metal MNs to generate reversible micro-channels in the SC. Drug-loaded nano-scale carriers are then applied, or permeation is further facilitated using other enhancement techniques, such as iontophoresis [19,20].

2,3,5,6-Tetramethylpyrazone (TMP), isolated from *Ligusticum wallichii* Franch., is widely used in China for the treatment of cardiovascular and cerebrovascular diseases [21]. Intravenous and peroral routes are favored for TMP administration, however, poor patient compliance, due to the long treatment period required (usually several months), compromises its effectiveness. TMP also has poor pharmacokinetic parameters, such as low and unstable oral bioavailability and a short half-life for elimination. Transdermal TMP administration has attracted increased attention, in part because of improved patient compliance associated with this route, but also due to the physical and chemical properties of TMP (small molecular weight and an ideal *n*-Octanol/water partition coefficient) [22,23,24]. Most investigations, however, have focused mainly on enhancements for passive skin penetration [25,26,27].

In this study, active (MN) and passive (ME) skin penetration enhancement techniques were combined to improve TMP skin permeation efficiency. To optimize the TMP-ME formulation, in vitro skin permeation experiments were conducted using a uniform experimental design, guided by a pseudo-ternary phase diagram. The effects of MN treatment on the skin permeation and transdermal absorption characteristics of TMP-ME were evaluated by in vitro skin permeation, in vivo skin microdialysis, and pharmacokinetic analysis in rats.

## 2. Results and Discussion

### 2.1. Optimization of Formulation for TMP-ME

**Figure 1:**
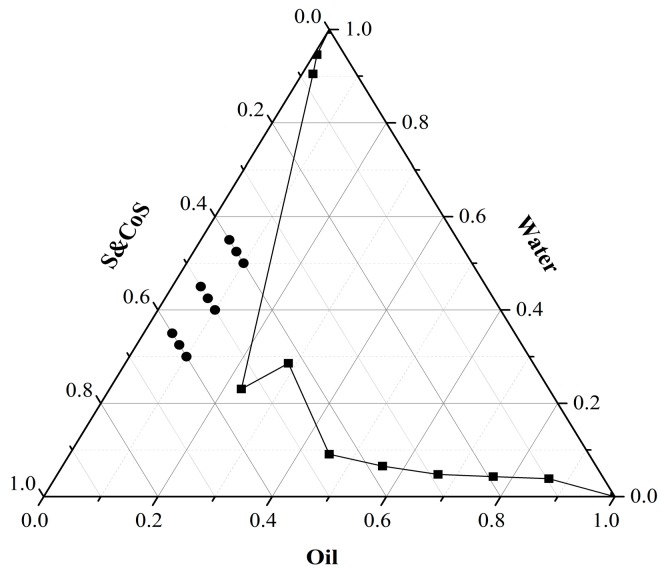
Pseudo-ternary phase diagram for TMP-ME. S&Cos is surfactant (Labrasol) and co-surfactant (Transcutol P); Oil is the oil phase (Maisine35-1); and circles show the compositions of TMP-MEs used for formulation optimization.

**Figure 2:**
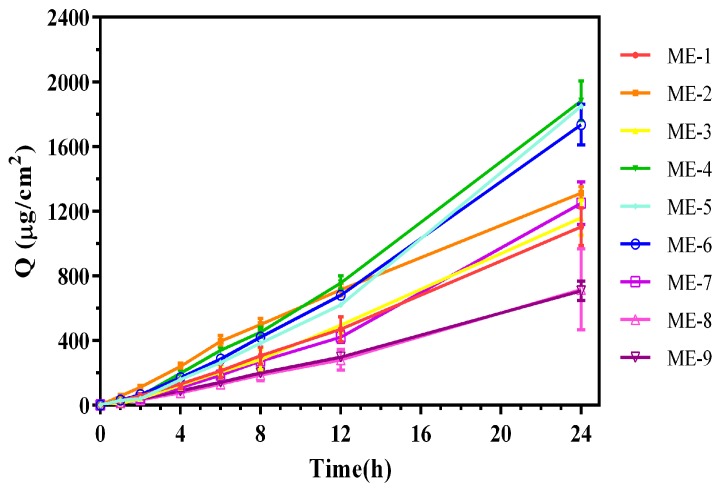
Cumulative in vitro skin permeation for TMP-MEs. The TMP-MEs used for formulation optimization are shown as ME-1 through ME-9.

The pseudo-ternary phase diagram for Maisine^®^ 35-1, Labrasol^®^, Transcutol^®^ P, and water is shown in Figure 1. To optimize the formulation, nine TMP-ME formulations were compared using a uniform experimental design guided by the pseudo-ternary phase diagram. The accumulation of TMP in skin in vitro is shown as a function of time in Figure 2 for each formulation, and TMP accumulation values and mean particle sizes are listed in Table 1.

Using these data, the mathematical functions relating TMP accumulation (*Y*_1_) or mean particle size (*Y*_2_) for TMP-ME, and the weight percentages of the oil phase (*X*_1_) and surfactant and co-surfactant (*X*_2_) components in TMP were modeled using multiple linear regression. The resulting equations are shown below:

*Y*_1_ = 2163.81*X*_1_ − 128.75*X*_2_ − 6.95*X*_1_*X*_2_ − 125.12*X*_1_^2^ + 1.75*X*_2_^2^ − 2815.02

*Y*_2_ = −33.137*X*_1_ − 14.626*X*_2_ + 0.389*X*_1_*X*_2_ + 1.888*X*_1_^2^ + 0.137*X*_2_^2^ + 470.191

The TMP-ME formulation was optimized using these functions. In the predicted optimum formulation, the weight percentages of the oil phase, surfactant, and co-surfactant were 7%, 30%, and 10%. The predicted accumulation of TMP over 24 h and the mean particle size were 1904.77 µg/cm^2^ and 73.82 nm. TMP-ME was then prepared using these parameters and analyzed as described earlier. The measured accumulation of TMP and the mean particle size were 2471.02 ± 41.57 µg/cm^2^ and 79.04 ± 0.68 nm, with the relative standard deviation (RSD) between predicted and measured values ranging from 28.11% to 32.11%, and 6.07% to 7.91%, respectively. These results suggest that the characteristics of TMP-ME can be reliably predicted using the established functions. The relatively high RSD observed between the predicted and measured values for TMP accumulation may be due to the intrinsically large experimental error in the in vitro experiment.

### 2.2. In Vitro Skin Permeation of MN-Assisted TMP-ME

**Figure 3:**
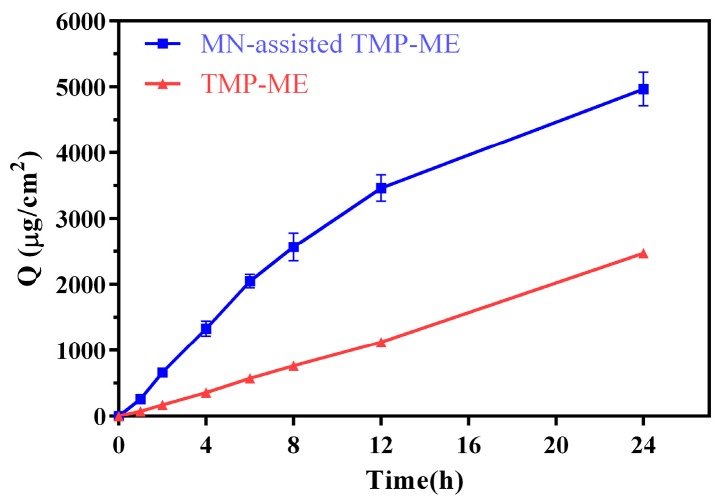
Cumulative in vitro skin permeation for TMP-ME and MN-assisted TMP-ME.

The cumulative permeation of TMP in skin in vitro is shown in Figure 3 for MN-assisted TMP-ME and TMP-ME alone. Compared with TMP-ME alone, a two-fold increase in cumulative permeation was observed for MN-assisted TMP-ME (2471.02 ± 41.57 µg/cm^2^ vs. 4963.61 ± 254.70 µg/cm^2^, *p* < 0.05). These results suggest that MN assistance can dramatically facilitate the penetration of skin by TMP-ME in vitro, consistent with the results obtained previously [28].

### 2.3. In Vivo Skin Microdialysis of MN-Assisted TMP-ME

**Figure 4:**
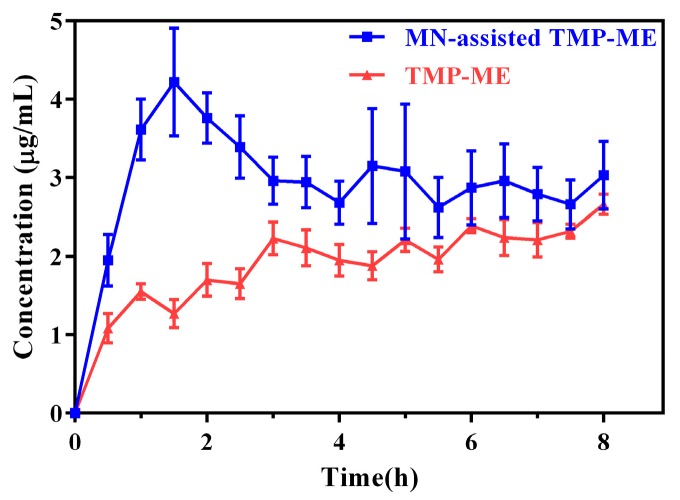
TMP levels in rat skin dialysate in vivo for TMP-ME and MN-assisted TMP-ME (*n* = 5).

To examine the influence of MN treatment on the ability of TMP-ME to penetrate skin, microdialysis experiments were conducted in vivo using rats. TMP levels in the recovered dialysate were measured over time for rats treated using MN-assisted TMP-ME or TMP-ME alone (Figure 4). The pharmacokinetic parameters for both treatments are listed in Table 2. Compared to rats treated with TMP-ME alone, a 1.25-fold increase in C_max_, a 0.93-fold decrease in T_max_, and a 0.88-fold increase in AUC_0–t_ were observed for TMP in rats treated with MN-assisted TMP-ME (*p* < 0.05). These results suggest that the rate and extent of percutaneous TMP absorption can be enhanced significantly by the application of MN-assisted TMP-ME, which is consistent with previous reports [29].

### 2.4. Pharmacokinetic Study of MN-Assisted TMP-ME

**Figure 5:**
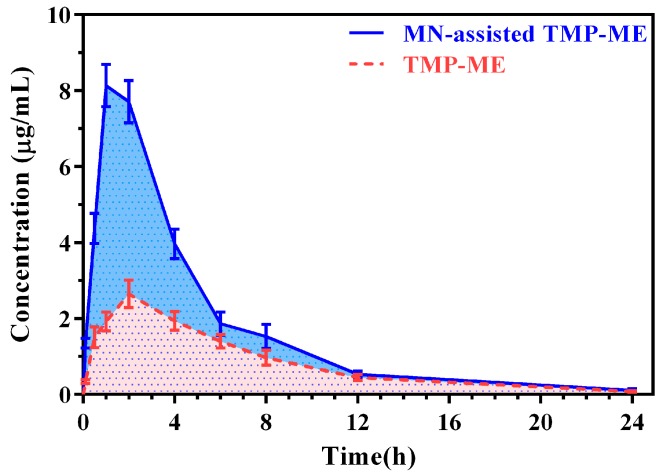
TMP levels in rat plasma for TMP-ME and MN-assisted TMP-ME (*n* = 5).

TMP levels in plasma for rats treated using MN-assisted TMP-ME and TMP-ME alone are shown in Figure 5. The corresponding pharmacokinetic parameters are listed in Table 3. Compared with TMP-ME alone, a 2.11-fold increase in C_max_, a 0.67-fold decrease in T_max_, and a 1.07-fold increase in AUC_0–t_ were observed for TMP in rats treated with MN-assisted TMP-ME (*p* < 0.05). These results suggest that the rate and extent of percutaneous absorption of TMP-ME can be enhanced dramatically buy MN assistance, consistent with our skin microdialysis experiments and previous reports [30].

### 2.5. Influence of MN Treatment on Transcutaneous Electrical Resistance

The transcutaneous electrical resistance of rat skin was measured in vitro before and after insertion of MN. The resistances were 850 ± 50 Ω/cm^2^ and 283 ± 104 Ω/cm^2^. respectively, suggesting that the skin barrier is severely compromised by treatment with MN. Our data are consistent with previous reports about the reversible reduction of skin integrity by MN [31,32].

## 3. Materials and Methods

### 3.1. Materials

2,3,5,6-Tetramethylpyrazone (TMP) was obtained from Nanjing Zelang Medical Technology Co., Ltd., Nanjing, China. Glyceryl monolinoleate (Maisine^®^ 35-1), caprylocaproyl macrogol-8 glycerides (Labrasol^®^), and highly-purified diethylene glycol monoethyl ether (Transcutol^®^ P) were purchased from Gattefossè, Saint Priest, France. Silicon MN (5 mm × 5 mm), with needles 250 μm long, 100 μm wide at the base, and spaced at 300 μm, were purchased from Jiangsu Natong Biological Co., Ltd. (Suzhou, China). Other chemicals are of HPLC or analytical grade.

### 3.2. Formulation Optimization of TMP-ME

The pseudo-ternary phase diagram for the oil phase, surfactant, and co-surfactant of TMP-ME was constructed using the water titration method as reported previously [33]. Briefly, the ratios of the weight of the oil phase (Maisine^®^ 35-1) relative to the weight of the mixture of surfactant (Labrasol^®^) and co-surfactant (Transcutol^®^ P) were 9:1, 8:2, 7:3, 6:4, 5:5, 4:6, 3:7, 2:8 and 1:9, with the K_m_ (the weight ratio of surfactant to co-surfactant) fixed at 3:1 (Table 4). Distilled water was then added drop-wise to these mixtures, and the weight ratios of oil phase, surfactant, co-surfactant, and water were recorded when the solutions changed from turbid from clear, and vice versa.

The formulation for TMP-ME was optimized using a uniform experimental design and the pseudo-ternary phase diagram. The weight content of Maisine^®^ 35-1 (*X*_1_), and Labrasol^®^ and Transcutol^®^ P (*X*_2_) were used as factors. The indices were the quantity of TMP that permeated the skin over a 24 h period (*Y*_1_), and the mean particle size of TMP-ME (*Y*_2_). 

The in vitro skin penetration behavior of the TMP-MEs was evaluated using a Franz-type diffusion cell. The effective penetration area was 1.7 cm^2^ and the receptor compartment volume was 16 cm^3^. Freshly-excised abdominal skin from male Sprague-Dawley (SD) rats was used as a permeation barrier. TMP-ME (1 mL) was added to the donor compartment. Thirty percent ethanol and 20% PEG-400 in normal saline (*v*/*v*) was added to the receptor compartment. At predetermined times, 1 mL of receptor medium was withdrawn, and replaced with the same volume of blank medium at 32 °C. The level of TMP in the receptor medium was determined using HPLC, and the total quantity that permeated the skin over a 24 h period was calculated as:

$$Qs=\frac{V}{S}\times C_{n}+\overset{n-1}{\underset{n-i}{\sum }}\frac{V_{i}}{S}C_{i}$$

In this expression, *C_n_* is the TMP concentration in the receptor medium at each sampling time, *C_i_* is the drug concentration of the sample, *V* and *V_i_* are the volumes of the receptor medium and the sample, and *S* is the effective diffusion area. 

Mean particle sizes for the TMP-MEs were measured using a Malvern Nano ZS90 Zetasizer (Malvern Instruments Ltd., Worcestershire, UK).

The mathematical functions relating indices and factors were established using a multiple linear regression method. On the basis of these functions, formulations for which the 24 h TMP permeation level exceeded 1900 µg/cm^2^ and the TMP-ME mean particle size was less than 75 nm were re-screened. *X*_1_ was varied from 5% to 10% (in 0.1% increments), and *X*_2_ was varied from 40% to 60% (in 1% increments).

In the optimized formulation of TMP-ME, the weight percentages of Maisine^®^ 35-1, Labrasol^®^, and Transcutol^®^ P were 7%, 30%, and 10%, respectively, and the loading of TMP was 1.5%. Triplicate TMP-ME samples were prepared using the optimum formulation. Mean particle size and TMP skin permeation over a 24 h period were measured and compared to the predicted values.

### 3.3. In Vitro Skin Permeation of MN-Assisted TMP-ME

The influence of MNs on the ability of TMP-ME to permeate skin was initially investigated using in vitro skin permeation experiments. For both TMP-ME alone and MN-assisted TMP-ME, experimental conditions and methods for TMP quantitation in the receptor medium were identical to those described above, except that the rat skin was pretreated with MN by manual insertion for 2 min in the MN-assisted TMP-ME analysis.

### 3.4. In Vivo Skin Microdialysis of MN-Assisted TMP-ME

Male SD rats were randomly assigned to either TMP-ME or MN-assisted TMP-ME groups (*n* = 5 for each). The skin microdialysis experiments for the TMP-ME group were conducted following methods described previously [34]. For the MN-assisted TMP-ME group, the protocol was identical, except that the rat skin was pretreated with MNs by manual insertion for 2 min. For both groups, rats were intraperitoneally anesthetized with urethane (1.3 g/Kg), and their abdominal hair was clipped. Microdialysis tubing (20 mm long, 200 µm inner diameter, 13,000 Da cut-off molecular weight) was inserted into the skin and perfused with 30% ethanol in phosphate-buffered saline (PBS) at 0.22 mL/h using a WZ-50C6 Micro Infusion pump (Smiths Medical, Norwell, MA, USA). an hour and a half after perfusion, 2 mL of TMP-ME was applied to the rat skin, and dialysate samples were collected from the tubes every 0.5 h for 8 h. TMP levels in the dialysate samples were determined using UPLC-MS/MS (Thermo Fisher Scientific Inc., Waltham, MA, USA).

### 3.5. Pharmacokinetics of MN-Assisted TMP-ME

The effect of MNs on the pharmacokinetic behavior of TMP-ME was assessed in male SD rats. The study design and treatments for the TMP-ME and MN-assisted TMP-ME groups were the same as in the skin microdialysis study. At 0.1, 0.5, 1, 2, 4, 6, 8, 12 and 24 h after the application of TMP-ME, 0.3 mL of blood was withdrawn from each rat. Plasma samples were immediately separated by centrifugation at 3000 rpm for 5 min. Plasma TMP levels were determined using UPLC-MS/MS. The non-compartmental pharmacokinetic parameters for TMP in both groups were calculated using BAPP2.0 (Bioavailability Program Package 2.0, Center for Metabolism and Pharmacokinetics, China Pharmaceutical University, Nanjing, China).

### 3.6. Influence of MN Treatment on Transcutaneous Electrical Resistance

The influence of MN treatment on skin barrier function was evaluated using transcutaneous electrical resistance [32]. Freshly-excised rat skin was mounted on a Franz-type diffusion cell, and PBS was used as the medium in both the donor and receptor compartments. Transcutaneous electrical resistance before and after MN insertion was measured in triplicate using Ag/AgCl electrodes.

## 4. Conclusions

The rate and extent of percutaneous absorption by TMP-ME can be significantly enhanced by MN assistance, which may be related to the reversible barrier perturbation effect of MN on skin.

## Figures and Tables

**Table 1:** Characteristics of TMP-ME formulations.

Formulation	TMP 24-h Cumulative Skin Permeation (µg/cm^2^)	Mean Particle Size (nm)
1	1103.90 ± 117.32	63.61 ± 0.72
2	1311.12 ± 40.24	86.84 ± 0.41
3	1158.98 ± 109.01	55.96 ± 0.34
4	1883.34 ± 123.26	101.2 ± 0.65
5	1849.33 ± 16.63	83.57 ± 0.59
6	1735.46 ± 125.78	96.04 ± 0.28
7	1248.86 ± 132.42	119.4 ± 0.15
8	714.97 ± 252.10	117.4 ± 0.35
9	1103.90 ± 117.32	189.2 ± 0.45

**Table 2:** TMP pharmacokinetic parameters for TMP-ME and MN-assisted TMP-ME in the skin microdialysis study.

Parameter	MN-Assisted TMP-ME	TMP-ME
C_max_ (µg/mL)	4.79 ± 0.68 *	2.13 ± 0.16
T_max_ (h)	1.60 ± 0.22 *	3.10 ± 0.22
AUC (h * µg/mL)	21.90 ± 2.14 *	11.65 ± 0.58

Note: * represents *p* < 0.05, compared with TMP-ME.

**Table 3:** TMP pharmacokinetic parameters for TMP-ME and MN-assisted TMP-ME in the pharmacokinetic study.

Parameter	MN-Assisted TMP-ME	TMP-ME
C_max_ (µg/mL)	8.25 ± 0.51 *	2.65 ± 0.36
T_max_ (h)	1.20 ± 0.45 *	2.00 ± 0.00
AUC (h * µg/mL)	41.11 ± 3.11 *	19.87 ± 2.40
t_1/2_ (h)	0.17 ± 0.02	0.16 ± 0.01
V (mL)	2219.01 ± 401.80 *	4642.66 ± 634.70
Cl (mL/h)	360.47 ± 28.82 *	746.00 ± 90.89
MRT (h)	4.56 ± 0.17 *	6.03 ± 0.33

Note: * represents *p* < 0.05, compared with TMP-ME.

**Table 4:** Experimental design for optimization of the TMP-MP formulation.

Formulation	Factor		
	*X*_1_ (%)	*X*_2_ (%)	Water (%)
1	1 (5.0)	4 (50.0)	45.0
2	2 (5.0)	8 (60.0)	35.0
3	3 (5.0)	3 (40.0)	55.0
4	4 (7.5)	7 (60.0)	32.5
5	5 (7.5)	2 (40.0)	52.5
6	6 (7.5)	6 (50.0)	42.5
7	7 (10.0)	1 (40.0)	50.0
8	8 (10.0)	5 (50.0)	40.0
9	9 (10.0)	9 (60.0)	30.0

Notes: *X*_1_ represents the weight content of Maisine35-1 (oil phase) in the TMP-ME formulation; *X*_2_ represents the weight content of Labrasol and Transcutol P (surfactant and co-surfactant); the loading of TMP is 1.5% (*w*/*w*).
